# Validation of the STOP-Bang questionnaire for screening of obstructive sleep apnea in the general population and commercial drivers: a systematic review and meta-analysis

**DOI:** 10.1007/s11325-021-02299-y

**Published:** 2021-01-28

**Authors:** Lina Chen, Bianca Pivetta, Mahesh Nagappa, Aparna Saripella, Sazzadul Islam, Marina Englesakis, Frances Chung

**Affiliations:** 1 Department of Anesthesia and Pain Management, Toronto Western Hospital, University Health Network, University of Toronto, Toronto, Ontario Canada; 2 Department of Anesthesia and Perioperative Medicine, London Health Sciences Centre and St. Joseph Health Care, Western University, London, Ontario Canada; 3 Library & Information Services, University Health Network, Toronto, Ontario Canada

**Keywords:** Obstructive sleep apnea, Screening questionnaire, STOP-Bang questionnaire, General population, Commercial drivers

## Abstract

**Purpose:**

Obstructive sleep apnea (OSA) is a critical occupational health concern, but is often undiagnosed in the general population and commercial drivers. The STOP-Bang questionnaire is a simple, reliable tool to screen for OSA, which could improve public health in a cost-effective manner. The objective of this systematic review and meta-analysis is to assess the validity of the STOP-Bang questionnaire to detect OSA in these key populations.

**Methods:**

We searched MEDLINE, Embase, Cochrane Central Register of Controlled Trials, Cochrane Database of Systematic Reviews, PsycINFO, Journals @ Ovid, Web of Science, Scopus, and CINAHL for relevant articles from 2008 to March 2020. The quality of studies was appraised using Cochrane Methods criteria. To calculate pooled predictive parameters, we created 2 × 2 contingency tables and performed random-effects meta-analyses.

**Results:**

Of 3871 citations, five studies that evaluated STOP-Bang in the general population (n = 8585) and two in commercial drivers (*n* = 185) were included. In the general population, prevalence of all OSA (AHI ≥ 5), moderate-to-severe OSA (AHI ≥ 15), and severe OSA (AHI ≥ 30) was 57.6%, 21.3%, and 7.8% respectively. In commercial drivers, the prevalence of moderate-to-severe OSA was 37.3%. The trends of high sensitivity and negative predictive value of a STOP-Bang score ≥ 3 illustrates that the questionnaire helps detect and rule out clinically significant OSA in the general population and commercial drivers.

**Conclusion:**

This meta-analysis demonstrates that the STOP-Bang questionnaire is a valid and effective screening tool for OSA in the general population and commercial drivers.

**Trial registration:**

PROSPERO No. CRD42020200379; 08/22/2020

**Supplementary Information:**

The online version contains supplementary material available at 10.1007/s11325-021-02299-y.

## Introduction

Obstructive sleep apnea (OSA) is characterized by cessation of breathing during sleep, which leads to poor sleep patterns and daytime somnolence. OSA is an increasingly common sleep-breathing disorder and a substantial public health concern [1, 2]. The reported prevalence of overall OSA in the general adult population ranges from 9 to 38% [3–5] with an estimated 80–90% of those individuals with OSA remaining undiagnosed [6, 7]. Among commercial drivers, who are a safety-sensitive occupational group, OSA is present in 24–28% of the workforce [8, 9]. If left undiagnosed and untreated, OSA can lead to serious health consequences including hypertension [10], cardiovascular diseases [11, 12], cognitive decline [13], depression [14], and all-cause mortality [15–17]. Furthermore, untreated OSA in non-commercial and commercial drivers has been strongly associated with an increased risk of motor vehicle accidents [18, 19]. Considering the significant public health and safety burden of unrecognized sleep apnea, early identification of OSA to initiate treatment is crucial. Although overnight laboratory polysomnography (lab PSG) is the gold standard for diagnosing OSA, it is time-consuming and costly. Portable or home sleep apnea testing (HSAT) may be more convenient but still requires the expertise of sleep medicine specialists for interpretation. Thus, a reliable screening tool that could facilitate early identification of at-risk individuals would be of tremendous help to healthcare professionals.

The STOP-Bang questionnaire screening tool is straightforward and self-reportable, and can be completed within 1 to 2 minutes [20, 21]. It is comprised of four self-reportable criteria (STOP: Snoring, Tiredness, Observed apnea, and high blood Pressure) and four demographic items (Bang: BMI, age, neck circumference, gender). If individuals score 3 affirmative answers or more, they are classified as being at risk of OSA [22]. If individuals score 5 affirmative answers or more, they are considered to be at high risk of OSA [22]. In the surgical setting, the sensitivity of a STOP-Bang score ≥ 3 is 84%, 93%, and 100% to predict all OSA (apnea-hypopnea index (AHI)≥ 5), moderate-to-severe OSA (AHI ≥ 15), and severe OSA (AHI ≥ 30), respectively [20]. Due to its practicality and high sensitivity, the STOP-Bang questionnaire has been validated in surgical and sleep clinic settings worldwide; however, its validity has not been reviewed in two important populations relevant to primary care and public health, which are the general population and commercial driver population [23, 24]. The objective of this systematic review and meta-analysis (SRMA) is to provide a comprehensive review of the predictive parameters of the STOP-Bang questionnaire in screening patients for OSA in the general population and commercial drivers. We hypothesize that the STOP-Bang questionnaire would be a useful armamentarium to screen for OSA in the general population and commercial drivers.

## Methods

### Literature search and study selection

The protocol of this SRMA was registered in the International Prospective Register of Systematic Reviews (PROSPERO) (CRD42020200379). We followed the Preferred Reporting Items for Systematic Reviews and Meta-analyses (PRISMA) guideline for this review [25]. A medical information specialist with expertise in systematic reviews (ME) designed and implemented the search strategy. The following databases were searched from January 2008 to March 2020 without language restrictions: MEDLINE, Medline-in-process, Embase, EmCare Nursing, Cochrane Central Register of Controlled Trials, Cochrane Database of Systematic Reviews, PsycINFO, Journals @ Ovid with full-text searching, all using the Ovid search interface; Web of Science (Clarivate Analytics), Scopus (Elsevier), and CINAHL. The search strategy included free-text and index terms: “stop-bang” or “stopbang”. A Web of Science citation search on the initial validation article for the STOP-Bang questionnaire was run to capture articles that cited it going forward in time [20]. Also, we performed a manual citation search to retrieve related articles and continued literature surveillance through August 2020. The full search strategies used are shown in Supplementary Digital Content (Supplementary Appendix 1).

### Selection of studies

After duplicates were removed, four reviewers (AS, SI, LC, BP) independently screened the titles and abstracts of retrieved articles using Rayyan [26]. Once irrelevant studies were excluded, full-text publications that met the following criteria were assessed for inclusion: (1) STOP-Bang questionnaire was evaluated in adults (age ≥ 18 years), in the general population or commercial drivers; (2) OSA diagnosis was validated against lab-PSG or HSAT, and (3) apnea-hypopnea index (AHI) or respiratory disturbance index (RDI) were used to define OSA and its severity. Studies with pediatric, veteran, and pregnant populations were excluded. Throughout this process, disagreements regarding inclusion of abstracts and full-text articles were resolved through discussion among the co-authors (LC, BP) and the senior author (FC).

### Data extraction and management

Two authors (LC, BP) independently recorded data from included studies using a pre-designed data collection form. The STOP-Bang score ≥ 3 was accepted as the threshold, and studies that only analyzed predictive parameters at other STOP-Bang thresholds were excluded. OSA was defined as AHI of ≥ 5 events per hour of sleep. Individuals with AHI ≥ 15 or RDI ≥ 15, and AHI ≥ 30 or RDI ≥ 30 were defined as having moderate-to-severe and severe OSA, respectively.

Predictive parameters reported at each AHI or RDI cutoff were used to compose a 2 × 2 contingency table for each study. If there was insufficient information to complete such table or if the study modified the STOP-Bang questionnaire, it was excluded.

### Assessment of methodological quality

Two reviewers (LC, BP) separately appraised the quality of included studies based on internal and external validity criteria described by the Cochrane Methods group on screening and diagnostic tests [27]. In the case of any disagreements, consensus was reached with a third reviewer (MN). We assessed internal validity through the following criteria: valid reference test, definition of disease, blind execution of the STOP-Bang questionnaire, independent interpretation of index test results from clinical information, and study design. We evaluated external validity using the following factors: disease spectrum, research setting, pre-screening or referral, availability of demographic information, explicit threshold of STOP-Bang, percentage of missing subjects, missing data management, and subject selection for PSG.

### Statistical analysis

By creating 2 × 2 contingency tables, the following paired and unpaired predictive parameters were recalculated with 95% confidence interval (CI): prevalence, sensitivity and specificity; positive predictive value (PPV) and negative predictive value (NPV); and diagnostic odds ratio (DOR). The area under the summary receiver operating characteristic curves (AUC) were calculated using logistic regression. We grouped studies according to the type of population (general population or commercial drivers) and the AHI cutoffs with reported validity parameters. We recalculated the pooled predictive parameters at each AHI severity cutoff, and composed forest plots with a random-effects model. To analyze the diagnostic accuracy of the STOP-Bang questionnaire, we performed AUC analysis. Heterogeneity or inconsistency was quantified using the chi-squared (chi^2^ or *X*^2^) test (*p* value < 0.05: heterogeneity present) and *I*^2^ test (*I*^2^ > 33%: heterogeneity present). Analyses were performed using Review Manager Version 5.4 Copenhagen (The Nordic Cochrane Centre, The Cochrane Collaboration, 2020) and MetaDisc Version 1.4 (Hospital Ramony Cajal, Madrid, Spain).

## Results

### Search results and selection process

**Fig. 1:**
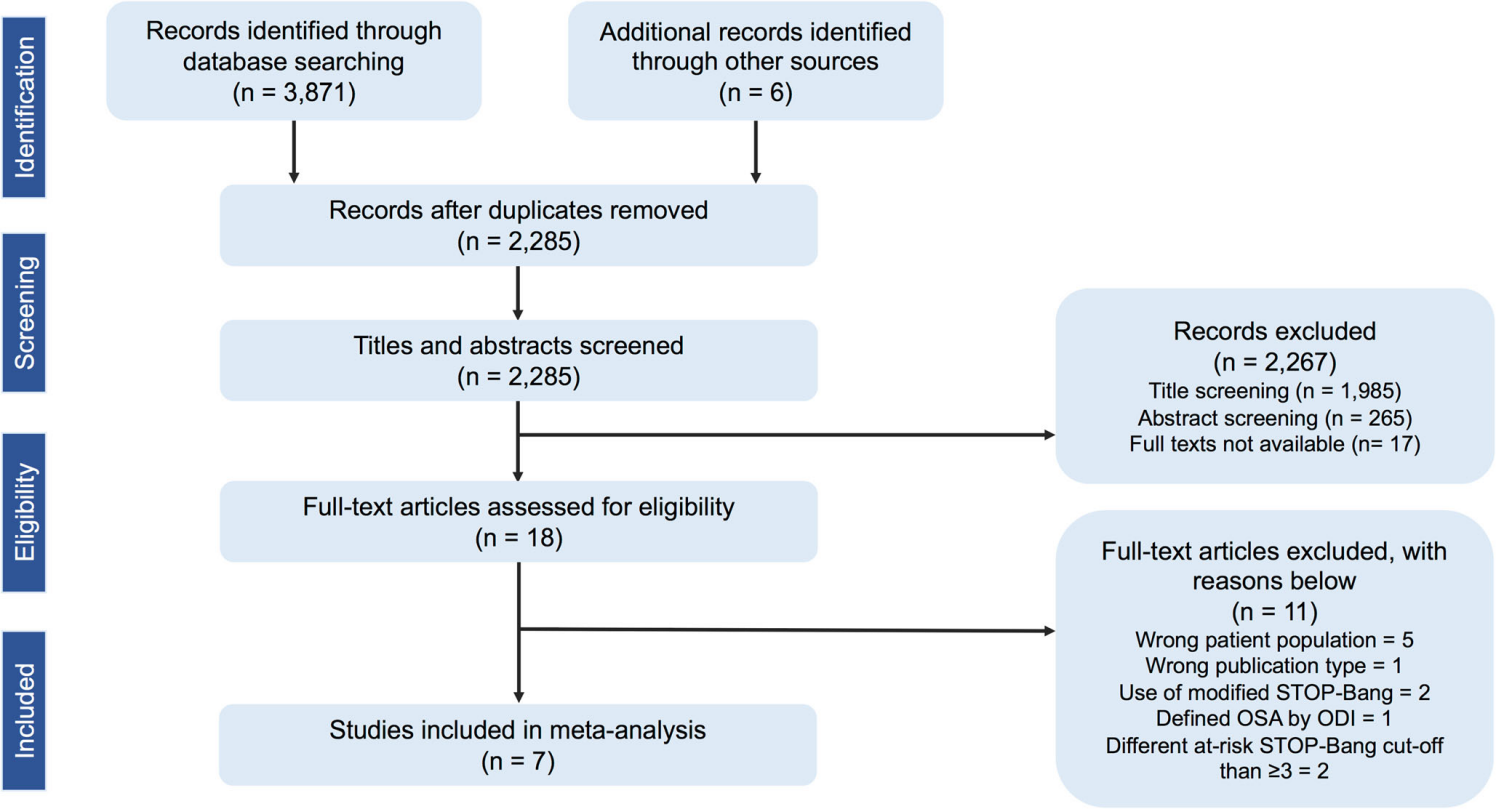
Flow diagram of search strategy used for systematic review and meta-analysis. *ODI*, oxygen desaturation index

Figure 1 showcases our literature search strategy, which initially yielded 3871 citations. We found six potentially relevant studies by citation search. Following removal of duplicates, 2285 studies were evaluated. After title and abstract screening, 2267 studies were excluded because predetermined eligibility criteria were not met. From the remaining 18 articles, we excluded 11 studies after full-text review for various reasons (Supplementary Table 1) [28–38]. Seven studies were included with a total of 8770 subjects: five in the general population (n = 8585) [39–43] and two in commercial drivers (n = 185) [44, 45]. The included studies were performed in various countries: the USA [39], Switzerland [40], Singapore [41], Chile [42], Belgium [43], Turkey [44], and Serbia [45].

From the general population, three studies (n = 3573) [40, 42, 43] were included for meta-analysis at AHI ≥ 5, five studies (n = 8586) [39–43] at AHI ≥ 15, and four studies (n = 8380) [39–41, 43] at AHI ≥ 30. Among commercial drivers, one study (n = 100) [45] evaluated diagnostic accuracy at AHI ≥ 5, two (n = 185) [44, 45] at AHI ≥ 15, and one (n = 100) [45] at AHI ≥ 30.

### Quality assessment of included studies

The results of internal and external validity assessment are presented in Supplementary Tables 2 and 3. With regard to internal validity, all selected studies used an accepted reference test to diagnose OSA and validate the STOP-Bang questionnaire. For blind execution of the index and reference tests, three studies were rated unclear risk of bias because they provided insufficient information as to whether the scoring of PSG readings and interpretation of STOP-Bang results were blinded. All seven studies had an unclear risk of bias regarding independent interpretation of the questionnaire to clinical information.

When evaluating external validity, all studies explicitly reported inclusion and exclusion criteria. Regarding pre-screening before application of STOP-Bang scoring, three studies stratified their subjects into risk groups and then applied the questionnaire [41, 42, 45]. Finally, four studies provided incomplete information and analysis of missing data describing basic characteristics of those who were not included, lost to attrition, etc. [39, 41, 43, 44]. Overall, all the studies had low-to-moderate risk of bias and were considered acceptable to answer the review question.

### Characteristics of included studies

The study characteristics and demographic data of the included studies are presented in Tables 1 and 2. Based on the available data, the general population had an average age of 60 ± 11 years with a mean BMI of 27 ± 5 kg/m^2^ and 51% were male. In contrast, the commercial drivers group had a mean BMI of 29 ± 5 kg/m^2^ and all participants were male. There were variations in the cutoff criteria for OSA: five studies defined OSA using AHI ≥ 5 [40–43, 45], and two as either AHI ≥ 15 [44] or RDI ≥ 15 [39]. The 2 × 2 contingency tables and predictive parameters for individual studies are shown in Supplementary Table 4.

**Table 1:** Characteristics of included studies in the general population and in commercial drivers

Study ID	Study population and sample size (*n*)	Study type and validation tool	OSA Prevalence (%)	OSA definition	No OSA AHI < 5 *n* (%)	Mild OSA AHI ≥ 5 to AHI < 15 *n* (%)	Moderate OSA AHI ≥ 15 to AHI < 30 RDI ≥ 15 to RDI < 30 *n* (%)	Severe OSA AHI ≥ 30 or RDI ≥ 30 *n* (%)
General population								
Silva [39] 2011	American 4770	Retrospective HSAT (Compumedics Portable PS-2)	19.9%	RDI ≥ 15	NA	RDI < 15: 3822 (80.1)	603 (12.7)	345 (7.2)
Marti-Soler [40] 2016	Swiss 1559	Prospective HSAT (Titanium Embla)	71.9%	AHI ≥ 5	438 (28.1)	570 (36.6)	333 (21.3)	218 (14.0)
Tan [41] 2016	Singaporean 242	Prospective HSAT (Embletta Gold)	72.7%	AHI ≥ 5	66 (27.3)	108 (44.6)	42 (17.4)	26 (10.7)
Saldías Peñafiel [42] 2019	Chilean 205	Cross-sectional HSAT (NA)	59.0%	AHI ≥ 5	84 (41.0)	67 (32.7)	33 (16.1)	21 (10.2)
Bauters [43] 2020	Belgian 1809	Prospective HSAT (ApneaLink)	45.1%	AHI ≥ 5	993 (54.9)	604 (33.4)	145 (8.0)	67 (3.7)
Commercial drivers								
Firat [44] 2012	Turkish 85	Cross-sectional lab PSG	54.1%	AHI > 15	NA	AHI < 15: 39 (46.9)	46 (54.1)	NA
Popević [45] 2017	Serbian 100	Prospective lab PSG lab (type III)	57.0%	AHI ≥ 5	43 (43.0)	34 (34.0)	11 (11.0)	12 (12.0)

*AHI* apnea-hypopnea index, *HSAT* home sleep apnea testing, *Lab* laboratory, *NA* not available, *OSA* obstructive sleep apnea, *PSG* polysomnography, *RDI* respiratory desaturation index

**Table 2:** Demographic data of individuals in the general population and in commercial drivers^a^

Study ID	No. of patients	Age (year)	Male (%)	BMI (kg/m^2^)	Neck circumference (cm)	STOP-Bang Score	AHI/RDI	Minimum SpO_2_ (%)
General population								
Silva [39] 2011	4770	62 ± 10	52	NA	NA	3.4 ± 1	NA	NA
Marti-Soler [40] 2016	2121 1559^†^	59 ± 11	48	26 ± 4	36.9 ± 4	NA	NA	NA
Tan [41] 2016	242	48 ± 14	50	26 ± 5	36.4 ± 4	2 ± 2	14 ± 14	83.5 ± 7
Saldías Peñafiel [42] 2019	205	51 ± 15	46	29 ± 5	37.9 ± 4	NA	NA	NA
Bauters [43] 2020	1809	56 ± 6	48	27 ± 5	37.5 ± 4	2 ± 2	NA	NA
Commercial drivers								
Firat [44] 2012	85	NA	100	29 ± 4	41.1 ± 3	NA	21 ± 17	NA
Popević [45] 2017	100	43 ± 11	100	29 ± 6	40.4 ± 3	NA	12 ± 15	NA

*AHI* apnea-hypopnea index, *NA* not available, *RDI* respiratory desaturation index

^a^Data are presented as mean ± SD when appropriate

^b^Demographic data was reported for 2121 total participants who underwent PSG, but STOP-Bang was validated in 1559 of 2121 individuals

### Predictive parameters in the general population

**Fig. 2:**
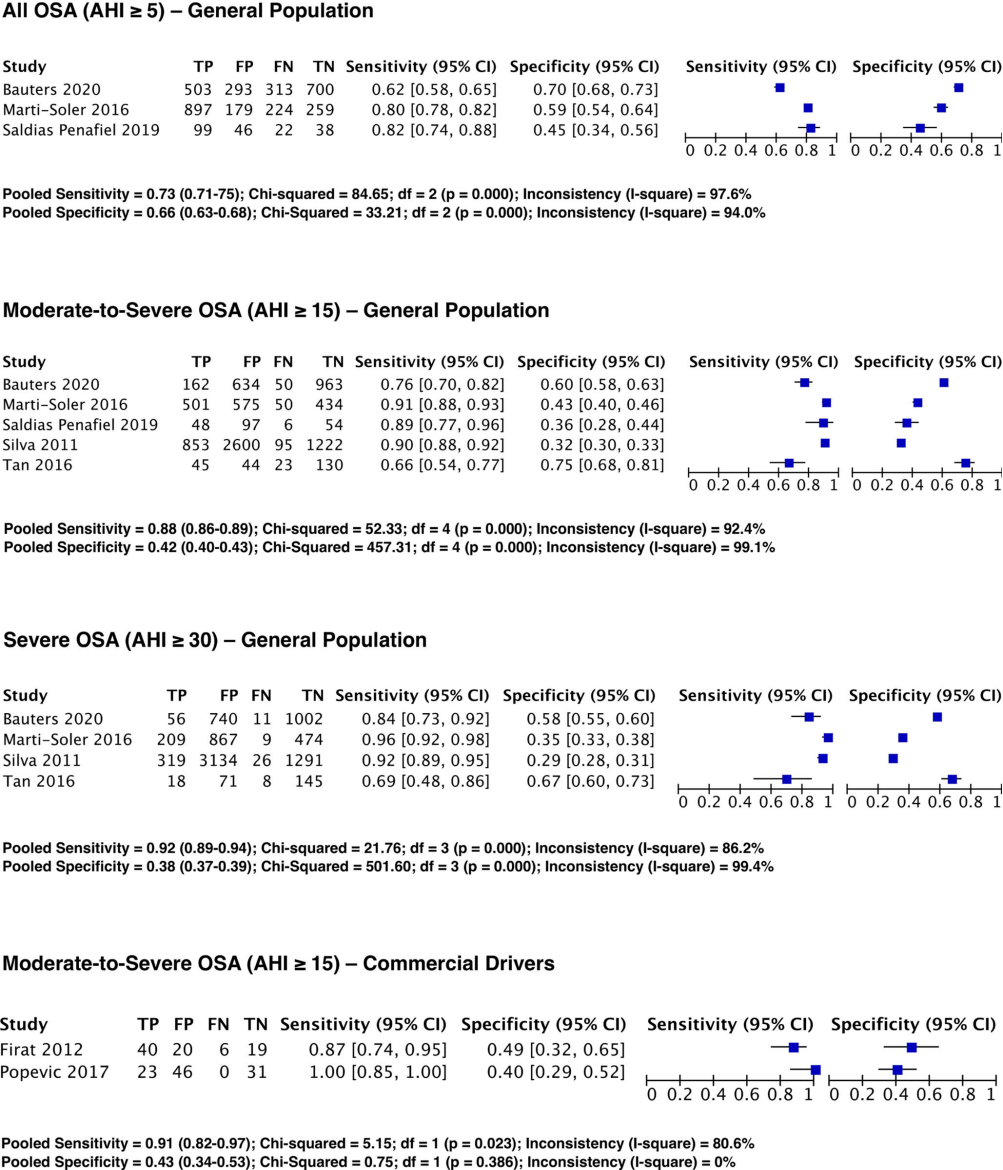
Forest plot for pooled sensitivity and specificity for various OSA severities in the general population and for moderate-to-severe OSA in commercial drivers. *TP*, true positive; *FP*, false positive; *FN*, false negative; *TN*, true negative; *CI*, confidence interval

The distributions of the pooled predictive parameters for a STOP-Bang ≥ 3 cutoff are summarized in Table 3 and Fig. 2. The prevalence of all OSA (AHI ≥ 5), moderate-to-severe OSA (AHI ≥ 15), and severe OSA (AHI ≥ 30) was 57.6%, 21.3%, and 7.8% respectively. A STOP-Bang score 3 or greater for an AHI cutoff ≥ 15 and 30 had excellent pooled sensitivities of 88% (95% CI: 86–89%) and 92% (95% CI: 89–94%), respectively. The STOP-Bang ≥ 3 also had high discriminative power to exclude moderate-to-severe and severe OSA as reflected by pooled NPVs (93% (95%CI: 92–94%) and 98% (95%CI: 98–99%), respectively). For the detection of all OSA at AHI ≥ 5, a STOP-Bang score ≥ 3 had moderate sensitivity (73%; 95%CI: 71–75%) and modest specificity (66%; 95%CI: 63–68%). The DOR increased with OSA severity and ranged from 4.5 (95%CI: 3.2–6.2) to 6.6 (95%CI: 4.3–10.3). The AUC was consistently > 0.73 for different severities of OSA with the highest for moderate-to-severe OSA at 0.76 (95% CI: 0.72–0.80).

**Table 3:** Pooled predictive parameters of STOP-Bang ≥ 3 as thresholds^a^

Predictive parameters (95% CI)	All OSA (AHI ≥ 5)	Moderate-to-severe OSA (AHI ≥ 15)	Severe OSA (AHI ≥ 30)
General population	3 studies; *n* = 3573	5 studies; *n* = 8586	4 studies; *n* = 8380
Prevalence	57 (56–59)	21 (20–22)	8 (7–8)
Sensitivity	73 (71–75)	88 (86–89)	92 (89–94)
Specificity	66 (63–68)	42 (40–43)	38 (37–39)
PPV	74 (72–76)	29 (28–30)	11 (10–12)
NPV	64 (62–67)	93 (92–94)	98 (98–99)
DOR	4.5 (3.2–6.2)	5.3 (4.1–6.9)	6.6 (4.3–10.3)
AUC	0.73 (0.71–0.75)	0.76 (0.72–0.80)	0.74 (0.72–0.76)
Commercial drivers	1 study; *n* = 100	2 studies; *n* = 185	1 study; *n* = 100
Prevalence	-	37 (30–44)	–
Sensitivity	-	91 (82–97)	–
Specificity	-	43 (34–53)	–
PPV	-	49 (40–58)	–
NPV	-	89 (77–95)	–
DOR	-	9 (2.1–37.6)	–

*OR* odds ratio, *CI* confidence interval, *AUC* area under summary of receiver operating characteristic curve

^a^Data are expressed as percentage and 95% confidence interval

### Predictive parameters in commercial drivers

In the commercial driver population, the prevalence of OSA for moderate-to-severe OSA was 37.3%. At this severity, a STOP-Bang score ≥ 3 shows excellent pooled sensitivity at 91% (95%CI: 82–97%) and pooled NPV at 89% (95%CI: 77–95%). The associated pooled specificity was 43% (95%CI: 34–53%) and PPV was 49% (95%CI: 40–58%) (Fig. 2, Table 3). The DOR was 9 (95%CI: 2.1–37.6) and an AUC curve could not be produced because of limited available studies.

## Discussion

This SRMA demonstrates that the STOP-Bang questionnaire is a valid screening tool in the general population and commercial drivers. The high sensitivity of a STOP-Bang score ≥ 3, ranging from 88 to 92%, helps to identify those in the community and commercial drivers who are at risk for moderate-to-severe and severe OSA. In the general population with a STOP-Bang score of 0–2, the high negative predictive values of 93% and 98% of a STOP-Bang score ≥ 3 means that we will be confident to rule out moderate-to-severe OSA and severe OSA. Similarly, the high NPV in commercial drivers of 89% also indicates that those with a STOP-Bang score of 0–2 have a low probability of having moderate-to-severe OSA. In the general population, the AUC is clinically significant (> 0.73) at each AHI threshold signifying the diagnostic utility of the STOP-Bang questionnaire. This trend in sensitivities and NPV is similar to STOP-Bang performance in different populations, such as the sleep clinic and surgical population [23].

There is usually a trade-off between the sensitivity and specificity of a screening tool so that the high sensitivity of the STOP-Bang questionnaire comes at the cost of high specificity. In this study, a STOP-Bang score ≥ 3 has relatively low to moderate specificity across all OSA severities and in both populations. This modest specificity may subject individuals to false-positive results, which leads to unnecessary PSG or HSAT testing. Nonetheless, minimizing false positives is of secondary importance when compared to the disease burden, safety hazard, and relatively higher cost of missed OSA cases [24, 46]. While the risk of further investigation if identified as positive by the STOP-Bang questionnaire is low, the risks associated with undiagnosed sleep apnea are dire [10–16, 18, 47]. For OSA, high sensitivity is clinically relevant as it enhances the early diagnosis of those with unrecognized OSA. Alternatively, a higher cutoff can be utilized which will increase specificity but lower sensitivity [21, 22]. Also, the STOP-Bang questionnaire can be used together with the Epworth Sleepiness Scale to increase specificity if needed [35].

### Application of the STOP-Bang questionnaire in the general population

We found a higher prevalence of OSA (58% at AHI ≥ 5) than past estimates [3]. This possible overestimation may be due to biased sampling methods from the general population. Silva et al. included older participants indirectly assembled from multiple longitudinal community-based cohorts [39], and Tan et al. used a sample of all snoring subjects and a sub-sample of non-snoring subjects from its existing population-based cohort [41]. Also, the high prevalence may be due to the increasing obesity epidemic, a strong risk factor for OSA [1, 48]. In addition, the changes in measurement techniques and scoring criteria for OSA may contribute to the higher prevalence of OSA [1, 48]. By using more recent OSA diagnosis criteria by the American Academy of Sleep Medicine, researchers have found prevalence of OSA which is comparable to the prevalence in our included studies [2].

For AHI ≥ 5 with high prevalence, the STOP-Bang questionnaire has moderate sensitivity and specificity at 73% and 66%, respectively. While AHI ≥ 5 is applicable for research purposes, this cutoff for mild-to-severe OSA is neither clinically significant nor strongly associated with adverse comorbidities [17]. Thus, we recommend the utilization of STOP-Bang questionnaire for its discriminative power for moderate-to-severe and severe OSA.

Often, primary care physicians have sufficient knowledge about OSA but fail to screen and manage their patients [49, 50]. Approximately 50% of general practitioners did not screen those at high risk for OSA, and 90% did not use OSA screening tools [51]. In general, the public has limited awareness of the presence of OSA and its devastating consequences [52, 53]. As many are asymptomatic and do not experience subjective sleepiness or impaired objective vigilance [54], individuals with undiagnosed OSA do not self-recognize their symptoms. Importantly, AHI does not necessarily correlate with extent of symptoms [55], which furthers the need for efficient screening and diagnosis. If primary care physicians suspect OSA, the STOP-Bang questionnaire helps with risk stratification and proper triage for appropriate care of OSA [56]. It reduces referral to overwhelmed sleep clinics with long wait times and is a feasible implementation process given the simplicity and short administration time of the questionnaire [57].

The American Academy of Sleep Medicine Clinical Practice Guidelines for diagnostic testing for OSA in adults recommended against the use of clinical tools, questionnaires, and prediction algorithms for the diagnosis of OSA [58]. The STOP-Bang questionnaire fulfills the unmet need for a screening test that effectively detects or rules out possible OSA in the community, and thus can save healthcare costs upstream of potential harms related to OSA. Its purpose is not to replace the PSG or HSAT.

### Utility of the STOP-Bang questionnaire in commercial drivers

Commercial drivers are mostly male, obese, and sedentary for extended periods of time, which are all significant risk factors for OSA [59, 60]. In this SRMA, the subjects were overweight with BMI < 30 kg/m^2^. The recent Canadian Clinical Guidelines on obesity in adults emphasize that obesity should be defined by how it impacts a person’s health as a chronic disease rather than by a misleading value such as BMI [48]. Furthermore, a bidirectional relationship exists between metabolic syndrome in male drivers and the presence of OSA [61]. These associations highlight the importance of screening commercial drivers for OSA, considering their potential comorbidities.

In occupations that require a high level of alertness, excessive daytime sleepiness caused by OSA poses serious risks for injuries and fatalities [62]. A study in heavy equipment operators found that higher AHI with sleepiness was significantly linked to more accidents [63]. Comparably in commercial airline pilots, nearly one-third of pilots were at high risk of OSA with half falling asleep without notifying their co-pilot [63, 64]. Untreated commercial drivers with severe OSA have a significantly increased risk of near-miss accidents and motor-vehicle accidents [19, 65]. For the safety of commercial drivers and those who share the road with them, it is imperative that commercial drivers be screened for OSA during medical examinations for their certification and at periodic intervals afterwards. In the USA, the Federal Motor Carrier Safety Administration (FMCSA) has yet to recommend the use of validated screening tools over traditional questioning by medical examiners [29, 66]. Commercial drivers may not be aware of OSA symptoms or may be reluctant to self-report OSA for reasonable fear of losing their licenses and experiencing financial hardship [19]. With its subjective and objective questions, the STOP-Bang questionnaire has a potential role in occupational clinical settings [60]. The STOP-Bang questionnaire rules out moderate-to-severe OSA confidently in commercial drivers who are considered low-risk by the questionnaire. Due to the limited studies, further research is recommended.

### Limitations

Our SRMA has some limitations. First, the studies in commercial drivers had small sample sizes which may have produced imprecise pooled predictive parameters with wide confidence intervals. Only male drivers were studied, so the results may not be applicable to female drivers. Second, the risk of bias in some studies remained unclear, and selection bias in the general population studies may have compromised external validity of the meta-analysis. Third, the inconsistency (I^2^) of the predictive parameters is consistently high presumably due to methodological heterogeneity. Another reason for the heterogeneity could be the variability in prevalence of OSA across the different populations and countries. Since heterogeneity was suspected, we used a random-effects model for meta-analysis. The attempted meta-regression analysis to explore the heterogeneity was not possible due to the limited number of studies in the meta-analysis. Fourth, our statistical method did not address the overestimation of overall diagnostic test accuracy resulting from the interpretation of each outcome measure individually. Hierarchical models, such as the bivariate random-effects model, could account for this limitation if there are more studies available for meta-analysis. Finally, all studies in the general population used HSAT as their reference test. Although HSAT is an accepted test for diagnosing OSA, there is uncertainty surrounding the time spent asleep which could lead to an underestimation of AHI [67]. Given the difficulty of administering PSG to the general population, we consider HSAT a reasonable reference test, especially since conducting the recording in the participant’s home can reduce information bias. Despite these limitations, our SRMA delivers an important interpretation of the available literature on STOP-Bang questionnaire in the general population and commercial drivers.

## Conclusions

In conclusion, this meta-analysis demonstrates that the STOP-Bang questionnaire has been validated to be a fast and easy-to-use screening tool for the general population and commercial drivers. At a score of 3 or greater, the STOP-Bang questionnaire performs with high sensitivity and NPV. Thus, primary care physicians and occupational safety examiners can confidently rule out clinically relevant OSA in their respective populations. From a public health perspective, wider use of the STOP-Bang questionnaire to screen for OSA helps improve public health and reduce safety risks caused by sleep apnea in the general population and especially in commercial drivers.

## Supplementary information

ESM 1 (PDF 154 kb)

## Data Availability

Not applicable.
