# Supplementary material for: Validation of the STOP-Bang questionnaire for screening of obstructive sleep apnea in the general population and commercial drivers: a systematic review and meta-analysis
Source: Sleep Breath. 2021 Jan 28;25(4):1741–51. doi: 10.1007/s11325-021-02299-y (PMC8590671; doi:10.1007/s11325-021-02299-y)
Supplement: Supplementary file 1 — (PDF 154 kb) [file 11325_2021_2299_MOESM1_ESM.pdf]

Supplementary Information (SI)

Validation of the STOP-Bang Questionnaire for screening of obstructive sleep apnea in the general population and commercial drivers: a systematic review and meta-analysis

Lina Chen BSc[1¶], Bianca Pivetta BSc[1¶], Mahesh Nagappa MBBS[2], Aparna Saripella MSc[1], Sazzadul Islam MSc[1], Marina Englesakis MLIS[3], Frances Chung MBBS[1]

[1]Department of Anesthesia and Pain Management, Toronto Western Hospital, University Health Network, University of Toronto, Toronto, ON, Canada
[2]Department of Anesthesia & Perioperative Medicine, London Health Sciences Centre and St. Joseph Health Care, Western University, London, ON, Canada
[3]Library & Information Services, University Health Network, Toronto, ON, Canada

All authors have [¶]These authors shared first authorship.

Corresponding Author: Frances Chung, Dept. of Anesthesia and Pain Medicine, University Health Network, University of Toronto, MCL 2-405, 399 Bathurst St. Toronto, ON, M5T2S8, Canada. Email: frances.chung@uhn.ca, 416 603 5118, Fax: 416-603-6494.

Appendix 1. Search history record for systematic review: STOP-BANG questionnaire screening tool

Review/Search Topic: STOP-BANG Questionnaire Screening Tool; years = >2008; no other limits applied	Searcher: Marina Englesakis
Investigator(s): Dr. Frances Chung, Rida Waseem, Anesthesia TWH	Date: Monday, March 30, 2020

Databases	Database Dates covered	Date Database was searched	# Citations	# Duplicate Citations	Total Citations remaining	Notes or Comments
Medline/MedlineALL (Ovid)	1946 – March 26, 2020	March 30, 2020	396			
Embase (Ovid)	1947 – March 27, 2020	March 30, 2020	763			
EmCare Nursing (Ovid)	1995 – present	March 30, 2020	9			
Cochrane Central Register of Controlled Trials (Ovid)	1991 – present	March 30, 2020	37			
Cochrane Database of Systematic Reviews (Ovid)	2005 – present	March 30, 2020	0			
APA PsycINFO (Ovid)	1806 – March Week #4, 2020	March 30, 2020	100			
Journals@Ovid via University of Toronto (full-text searching)	March 30 2020	March 30, 2020	689			
Web of Science (Clarivate) including citation search (Chung, Anesthesiology 2008, 812)	1900 – March 27, 2020	March 30, 2020	1127			
Scopus (Elsevier)	1960 – present	March 30, 2020	474			
CINAHL with Full Text (EbscoHost)	1982 – present	March 30, 2020	276			
		Totals:	3871	0	0	Results in a compressed EndNote Library

Supplementary Table 1. Excluded studies and reasons for exclusion

Study ID	Use of STOP-Bang (SB)	n	High risk STOP-Bang ≥ 3	Low risk STOP-Bang 0-2	Reasons for exclusion
Kunisaki[36] 2014	Referred to sleep clinic from Veteran Affairs health care provider	American 1196	1170	26	*Wrong patient population*
Lockhart[37] 2015	Volunteer from outpatient clinics or inpatient antepartum obstetric service	American 248	48	200	*Wrong patient population*
Tantrakul[38] 2015	High-risk pregnancy clinic	Thai 72	19	53	*Wrong patient population and BMI > 27.5*
Evans[29] 2017	Commercial drivers presenting for DOT (Department of Transportation) physical examinations	American 12	11	1	*Wrong publication type:* a quality improvement project including validation with small sample size
Nahapetian[33] 2017	Tested weighted STOP-Bang in derivation and validation groups from the Sleep Heart Study	American 4774	NA	NA	*Wrong patient population:* same population as Silva[39] 2011 and inadequate information
Rebelo-Marques[34] 2017	Validated STOP-Bang in patients referred to sleep clinic from primary care	Portuguese 259	243	16	*Wrong patient population:* patients had suspected sleep disorders
Cruces-Artero[28] 2019	Multicenter, primary care: convenience sampling	Spanish 178	65	113	*Different high-risk STOP-Bang cut-off:* STOP-Bang ≥ 4 for females and ≥ 6 for males
Jeon[30] 2019	Validated STOP-Bang in community-dwelling sample recruited through advertisements	Korean 116	50	66	*Use of modified STOP-Bang:* BMI > 30
Senaratna[35] 2019	Validated STOP-Bang in randomly invited subjects from the Tasmanian Longitudinal Health Study, with OSA symptoms	Australian 286	206	80	Use of ODI (oxygen desaturation index) instead of AHI
Martins[31] 2020	Validated STOP-Bang in community-dwelling adults aged 65+	Brazilian 458	417 (≥2)	41 (<2)	*Different high-risk STOP-Bang cut-off*: STOP-Bang ≥ 2 because all subjects were 50+ years old
Massongo[32] 2020	Validated STOP-Bang in randomly selected community sample	Cameroonian 102	74	28	*Use of modified STOP-Bang:* BMI > 30

Supplementary Table 2. Appraisal of the included studies based on criteria for internal validity

Internal Criteria	Valid reference standard	Definition of the disease based on reference standard	Blind execution of index test and reference test	Index test interpreted independently of clinical information	Study design
**Definition**	**Laboratory PSG or Home Sleep Apnea Test (HSAT)**	**OSA diagnosed based on the PSG results (F)**	**PSG readings blinded to the questionnaire results and vice versa (F)**	**The questionnaire interpreted independently of clinical information (F)**	**Prospective or Retrospective**
General population					
Silva[39] 2011	HSAT	F	U	U	Retrospective
Marti-Soler[40] 2016	HSAT	F	F	U	Prospective
Tan[41] 2016	HSAT	F	F	U	Prospective
Saldías Peñafiel[42] 2019	HSAT	F	F	U	Prospective
Bauters[43] 2020	HSAT	F	F	U	Prospective
Commercial Drivers and Pilots					
Firat[44] 2012	Lab PSG	F	F	U	Prospective
Popević[45] 2017	Lab PSG, HSAT in lab	F	U	U	Prospective

F: Full meeting criteria; P: Partially meeting criteria; U: Unsure if meeting criteria in subgroups; not sure; N: Not meeting criteria in subgroups; N/A: Not applicable

Supplementary Table 3. Appraisal of the included studies based on criteria for external validity

External Criteria	Spectrum of diseases	Settings	Previous screening	Demographic information	Explication of cut-off point of index test	Percentage missing	Missing data management	Subject selection for reference test
**Definition**	**Inclusion and exclusion criteria mentioned (F)**	**Enough information to identify setting (F)**	**No pre-screening before application of the questionnaire (F)**	**Age, gender, BMI data provided (F)**	**Results presented for AHI ≥5 or RDI≥15 (F)**	**Percentage missing mentioned (F)**	**Analysis of missing data for basic characteristics (F)**	**All subjects were invited or randomly selected to do PSG (F)**
**General Population**								
Silva[39] 2011	F	F	F	F	F	F	U	F
Marti-Soler[40] 2016	F	F	F	F	N	F	F	F
Tan[41] 2016	F	F	N	F	F	F	P	F
Saldías Peñafiel[42] 2019	F	F	P	F	F	F	F	F
Bauters[43] 2020	F	F	F	F	F	F	P	F
Commercial Drivers and Pilots								
Firat[44] 2012	F	F	F	P	F	F	N	F
Popević[45] 2017	F	F	N	F	F	N/A	N/A	F

F: Full meeting criteria; P: Partially meeting criteria; U: Unsure if meeting criteria in subgroups; not sure; N: Not meeting criteria in subgroups; N/A: Not applicable

Supplementary Table 4. Tables describing 2x2 contingency values and predictive parameters of individual studies for all OSA (AHI ≥ 5), moderate-to-severe OSA (AHI ≥ 15) and severe OSA (AHI ≥ 30) in the general population and commercial drivers

4A: Description of 2x2 contingency table

	Polysomnography Positive (PSG +)	Polysomnography Negative (PSG -)	Total
**STOP-Bang Positive (SB+)**	True Positive	False Positive	
**STOP-Bang Negative (SB-)**	False Negative	True Negative	
**Total**			

4B: 2x2 contingency table for General Population – All OSA or AHI ≥ 5

Author	True Positive	False Positive	False Negative	True Negative	Sensitivity (95% Confidence Interval)	Specificity (95% Confidence Interval)
**Marti-Soler[2016]**	897	179	224	259	0.80 [0.78, 0.82]	0.59 [0.54, 0.64]
**Saldías Peñafiel[2019]**	99	46	22	38	0.82 [0.74, 0.88]	0.45 [0.34, 0.56]
**Bauters[2020]**	503	293	313	700	0.62 [0.58, 0.65]	0.70 [0.68, 0.73]

4C: 2x2 contingency table for General Population – Moderate-to-Severe OSA or AHI ≥ 15

Author	True Positive	False Positive	False Negative	True Negative	Sensitivity (95% Confidence Interval)	Specificity (95% Confidence Interval)
**Silva[2011]**	853	2600	95	1222	0.90 [0.88, 0.92]	0.32 [0.30, 0.33]
**Marti-Soler[2016]**	501	575	50	434	0.91 [0.88, 0.93]	0.43 [0.40, 0.46]
**Tan[2016]**	45	44	23	130	0.66 [0.54, 0.77]	0.75 [0.68, 0.81]
**Saldías Peñafiel[2019]**	48	97	6	54	0.89 [0.77, 0.96]	0.36 [0.28, 0.44]
**Bauters[2020]**	162	634	50	963	0.76 [0.70, 0.82]	0.60 [0.58, 0.63]

4D: 2x2 contingency table for General Population – Severe OSA or AHI ≥ 30

Author	True Positive	False Positive	False Negative	True Negative	Sensitivity (95% Confidence Interval)	Specificity (95% Confidence Interval)
**Silva[2011]**	319	3134	26	1291	0.92 [0.89, 0.95]	0.29 [0.28, 0.31]
**Marti-Soler[2016]**	209	867	9	474	0.96 [0.92, 0.98]	0.35 [0.33, 0.38]
**Tan[2016]**	18	71	8	145	0.69 [0.48, 0.86]	0.67 [0.60, 0.73]
**Bauters[2020]**	56	740	11	1002	0.84 [0.73, 0.92]	0.58 [0.55, 0.60]

4E: 2x2 contingency table for Commercial Drivers– All OSA or AHI ≥ 5

Author	True Positive	False Positive	False Negative	True Negative	Sensitivity (95% Confidence Interval)	Specificity (95% Confidence Interval)
**Popević[2017]**	49	20	8	23	0.86 [0.74, 0.94]	0.53 [0.38, 0.69]

4F: 2x2 contingency table for Commercial Drivers– Moderate-to-Severe OSA or AHI ≥ 15

Author	True Positive	False Positive	False Negative	True Negative	Sensitivity (95% Confidence Interval)	Specificity (95% Confidence Interval)
**Firat[2012]**	40	20	6	19	0.87 [0.74, 0.95]	0.49 [0.32, 0.65]
**Popević[2017]**	23	46	0	31	1.00 [0.85, 1.00]	0.40 [0.29, 0.52]

4G: 2x2 contingency table for Commercial Drivers– Severe OSA or AHI ≥ 30

Author	True Positive	False Positive	False Negative	True Negative	Sensitivity (95% Confidence Interval)	Specificity (95% Confidence Interval)
**Popević[2017]**	12	57	0	31	1.00 [0.74, 1.00]	0.35 [0.25, 0.46]
